# Research on a Novel Locating Method for Track Inspection Based on Onboard Sensors in Maglev Train

**DOI:** 10.3390/s21093236

**Published:** 2021-05-07

**Authors:** Yihong Yuan, Yanyun Luo, Feng Ye, Zhiwei Zhu, Guofeng Zeng, Guoqiang Wang

**Affiliations:** 1 National Maglev Transportation Engineering R&D Center, Tongji University, Shanghai 201804, China; yuanyihong@tongji.edu.cn (Y.Y.); zhuzhiwei@tongji.edu.cn (Z.Z.); zengguofeng@tongji.edu.cn (G.Z.); wangguoqiang@tongji.edu.cn (G.W.); 2 Institute of Railway and Urban Mass Transit, Tongji University, Shanghai 201804, China; yanyunluo@tongji.edu.cn

**Keywords:** maglev train, guideway inspection, locating

## Abstract

Guideway inspection is of great significance to the operation safety and riding quality of a commercial high-speed maglev transportation system. When analyzing guideway inspection data, it is important to obtain the location information for each piece of raw data and convert it from the time domain to the spatial domain for the analysis afterward. Previous studies have used the method of adding additional hardware such as GPS (global positioning system) receivers, LRF (location reference flag) readers, or onboard CAN (controller area network) bus adaptors to obtain location information. This paper presents a novel method for indirectly obtaining the location information via the use of data from the levitation and guidance control sensors perpendicular to the longitudinal direction to extract the characteristic information from the track. The method can be used for a long stator linear motor-driven maglev system and similar contactless rail transit systems. The results showed that the method could accurately identify the required location information in each stator tooth during the entire operation simultaneously with the operating information such as train velocity, direction, and track ID, without additional hardware installation and vehicle network connection. Thus, it could improve the pertinence of the results of guideway inspection equipment, and at the same time, facilitate the miniaturization and independence of guideway inspection equipment.

## 1. Introduction

**Figure 1:**
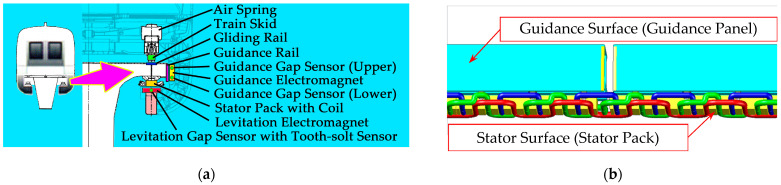
(**a**) Schematic diagram of the Transrapid maglev system; (**b**) surfaces of the maglev track.

Transrapid is a kind of electromagnetic levitation rail transit system. It uses constant conductor electromagnets to attract the track to achieve the suspension and guidance of the train; the main composition of the system is shown in Figure 1a. Through active control, a gap of 8–10 mm between the train electromagnet and the track is always maintained, and the long stator synchronous linear motor realizes the traction and braking of the train. The system achieves a completely noncontact operation at high speed. As an example, the Shanghai high-speed maglev demonstration line [1] achieved a commercial operating speed of 430 km/h.

For commercial high-speed maglev transportation systems, guideway inspection is of great significance to the operation safety and riding quality. To improve maintenance efficiency, guideways are inspected online by special guideway inspection equipment installed on the passenger train. The equipment evaluates irregularities at both the stator surface and the guidance surface (as shown in Figure 1b) by collecting levitation and guidance gap signals shared by gap sensors used for levitation and guidance electromagnet control.

When analyzing the track inspection data of the stator surface and the guidance surface, each piece of data collected by the track inspection equipment in the time domain needs to be located and converted to the spatial domain for further analysis. The locating accuracy required is determined by the inspection content: the data analysis of long-wave deviations requires track-beam-level locating with an effective resolution of about 25 m, while short-wave deviations require stator-tooth-level locating with an effective resolution of about 86 mm.

In the traditional wheel-rail railway field, locomotive and track inspection vehicles usually use incremental rotary encoders to obtain the speed and the locating information. The encoder is installed at the end of the wheel axle to collect the rotation angle of the axle. By counting the pulses generated from the encoder, one can easily obtain the rotation angle of the wheel. Then, the relative mileage can be calculated based on the wheel diameter. In railway vehicles, the wheel diameter is a variable that needs to be dynamically calibrated, as it can be affected by factors such as wheel wear and tread taper.

In the maglev field, the noncontact design implies that rotary encoders cannot be used as locating sensors; only contactless sensors can be introduced. Current vehicle-mounted maglev speed measuring and locating systems are divided into two types. One is the system used for the Operation Control System (OCS), whereas the other is an additionally installed locating system used for track inspection equipment. The former usually uses relative positioning sensors and absolute positioning sensors independently and redundantly installed at both ends of the train to collect locating information [1,2,3]. The relative position sensor obtains the relative mileage pulse signal and the phase signal for linear motor control by sensing the periodic feature structure of the track, such as the tooth-slot of the stator iron core [4,5,6,7,8], the metal sleeper [9], or the cross-inductive loop cable [10,11,12]. The absolute positioning sensor reads the coded information embedded in the passive location reference flags (LRFs) fixed at specific positions on the track through multiple pairs of transceiver coils arranged in the U-shaped sensor to obtain the absolute position information of the vehicle to eliminate the accumulated mileage error caused by various reasons from the relative position sensor [13,14].

This type of locating system can continuously output the position information of the train, it has obvious advantages in real-time, it is reliable, and it is suitable for operation control systems and vehicle control systems. However, this system uses multiple longitudinally redundant dedicated sensors that need extra hardware channels, and there may be cumulative errors in the mileage between two adjacent LRF boards, so it is not suitable for independent onboard guideway inspection equipment.

The locating methods used in traditional guideway inspection equipment include: the installation of an extra GPS receiver to obtain spatial position information at low accuracy (accurate to track beam) but high operating speed (above 400 km/h) requirements [15,16]; the installation of extra measuring wheels with a rotary incremental encoder to obtain mileage at high accuracy (accurate to stator pack or tooth-slot) but low operating speed (below 50 km/h) requirements [17,18,19]; the introduction of external information sources such as vehicle CAN networks and LRF sensor signals at both high accuracy and high operating speed requirements [20,21,22]. All the above methods require the addition of extra hardware equipment and data channels, which will not only increase the complexity of the system, but also reduce the independence of track inspection equipment.

At present, there is no locating method suitable for track inspection equipment to work at high speed (above 400 km/h) with high precision (stator pack level or higher) without introducing CAN information and LRF information.

Multi-sensor fusion (MSF) technology is a computer-based information processing technology that has emerged in recent years. It automatically analyzes and synthesizes information and data from multiple different signal sources (sensors) under certain rules in order to complete the required decisions and estimations. At present, it is mainly used in industries such as aircraft navigation, autonomous driving, robot control, image analysis, and complex industrial process control [23,24,25]. It is suitable for processing sensor signals of different types, different moments, different positions, and different credibilities. A study in the literature [26] proposed a speed measuring and locating method based on Kalman filter fusion using the combination of Beidou satellite positioning, inertial measurement units (IMU), and Doppler radar, and the study conducted a simulation at a speed of 120 km/h. This method can be used for relatively independent guideway inspection equipment, but it also requires additional hardware such as Doppler radar and Beidou satellite navigation equipment.

This paper takes the data collected by the maglev Track Inspection System TIS [27,28,29], developed by the National Maglev Transportation Engineering R&D Center (NMTC), as an example to carry out the research, referring to the idea of multi-sensor fusion to attempt to obtain high-precision positioning information from the track characteristic contained in the signals of different guideway inspection sensors at high speeds without adding additional hardware.

## 2. Signal Characteristics of Guideway Inspection Sensor

**Figure 2:**
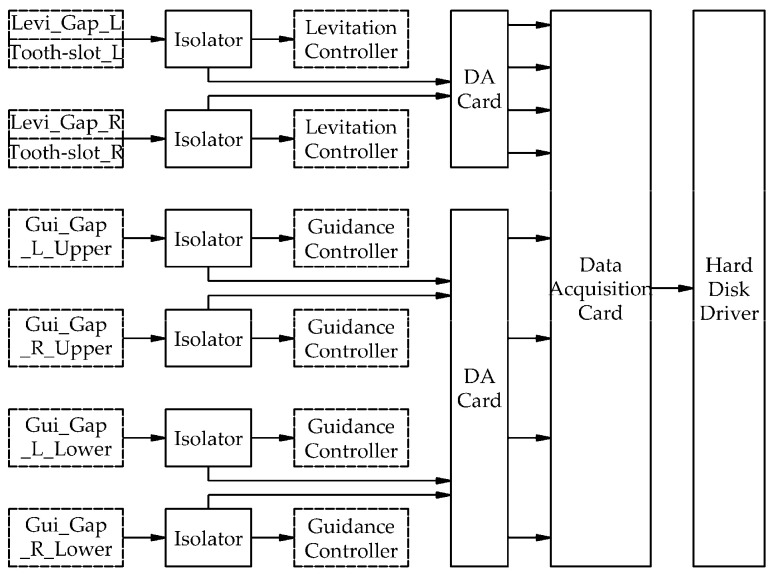
Data collection flowchart.

Guideway inspection equipment collects data from gap sensors (as shown by the blue dots in Figure 1) for levitation and guidance control systems, as required. The data include levitation gap signals and tooth-slot signals from levitation gap sensors and guidance gap signals from guidance gap sensors, with a total of eight channels (as shown in Table 1). The data are collected by an acquisition card after being isolated and converted to analog signals by a special adapter card, as shown in Figure 2. In the figure, all the components inside the solid boxes were newly installed for guideway inspection, while all the components inside the dashed boxes, including all the sensors, were originally present on the train. No new sensors were needed. The sampling method was equal-interval sampling in the time domain, the sampling rate was 10 kHz, and the interval between two samples was 0.1 ms. The description of each signal is as follows:

### 2.1. Levitation Gap Signal

**Figure 3:**
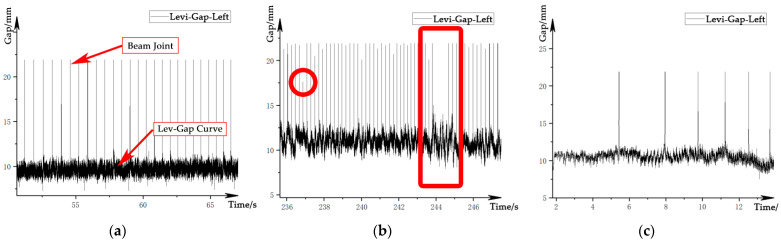
Waveform of the levitation gap signal: (**a**) ideal signal; (**b**) structure-affected signal; (**c**) velocity-affected signal.

The levitation gap signal is a signal that characterizes the distance between the levitation electromagnet of the train and the stator surface of the track, which is used for levitation control of the train. The signal is measured by an eddy current sensor per side. When the gap sensor passes through the track beam joint, the output signal becomes saturated due to the short-term loss of the measured surface, which is reflected in the data as a peak with an amplitude of about 23 mm, as shown in Figure 3a. The amplitude of the beam-end effect shows a certain randomness with the increase in vehicle speed, as shown by the circle in Figure 3b. In extreme cases, the peak may be overwhelmed by the gap fluctuation below. The gap also changes irregularly with the arrangement of the track beam, as shown by the rectangle in Figure 3b. In addition, the waveform of the gap signal is also affected by the speed of the train, as shown in Figure 3c.

### 2.2. Guidance Gap Signal

**Figure 4:**
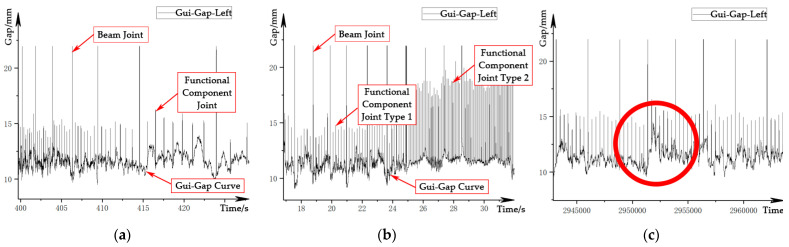
Waveform of the guidance gap signal: (**a**) velocity-affected signal; (**b**) structure-affected signal; (**c**) irregularity-affected signal.

The guidance gap signal is a signal that characterizes the distance between the guidance electromagnet of the train and the guidance surface of the track, which is used for the guidance control of the train. The signals are measured by two eddy current sensors per side. The time-domain waveform of the guidance gap signal is shown in Figure 4. The signal peaks both at the beam joints and functional component joints, while the amplitude of the latter is lower than that of the former. The amplitude also changes due to the influence of the track structure, as shown in Figure 4b, and in extreme cases, it becomes overwhelmed by gap fluctuations caused by track irregularities, as shown in Figure 4c, so it is not easy to identify. In addition, due to the limitation of the vehicle structure, the guidance gap sensors and the levitation gap sensors cannot be installed in the same section. This leads to an extra phase difference of the signal in the spatial domain.

### 2.3. Tooth-Slot Signal

**Figure 5:**
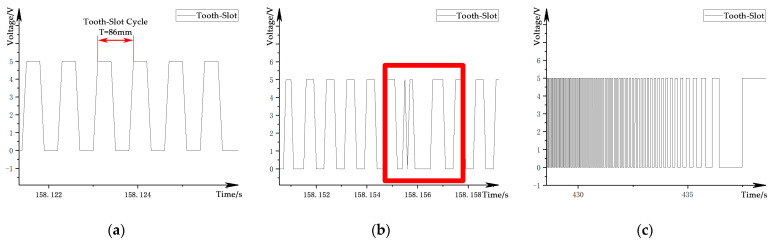
Waveform of the tooth-slot signal: (**a**) ideal signal; (**b**) structure-affected signal; (**c**) speed-affected signal.

The tooth-slot signal is a square wave speed pulse signal from the levitation gap sensor for auxiliary levitation control, one channel for each side. This signal is different from the relative position sensor used for OCS, and it cannot provide continuous phase information. The physical period of the tooth-slot signal is 86 mm in the spatial domain, as shown in Figure 5a. Unlike the ideal square wave signal obtained by traditional wheel-rail trains or road vehicles using a rotary incremental encoder at the end of the wheel shaft, the tooth-slot signal is affected by the arrangement of the stator pack, and there will be interference near the track beam joint, as shown by the rectangular area in Figure 5b. At the same time, the train speed will also affect the tooth-slot frequency in the time domain, as shown in Figure 5c.

### 2.4. Comparison of the Signals

A summary of the information contained in the three sensor signals is shown in Table 2:

In summary, all three kinds of signals contain certain position information, but with quasi-random interference. Therefore, the position information cannot be obtained reliably and accurately by using any signal alone.

## 3. Method for Locating from the Guideway Inspection Data

**Figure 6:**
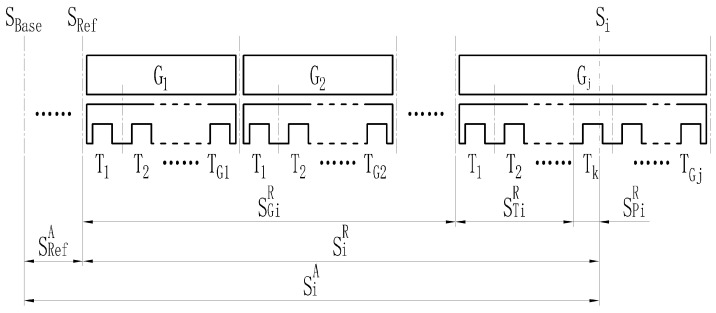
Schematic diagram of locating principle.

According to the characteristics of the maglev track and the signal from the guideway inspection sensors, the locating method based on the long stator tooth-slot arrangement was designed as follows:

Taking the forward running at the left side of the track as an example, as shown in Figure 6, $G_{1}$…$G_{j}$ are the track beams and $T_{1}$…$T_{Gj}$ are the stator teeth on the track beam $G_{j}$; $S_{Base}$ is the origin of the line’s absolute space mileage, $S_{Ref}$ is the reference point for relative space mileage conversion on the route; superscript *A* is the absolute space mileage of the route based on $S_{Base}$, while superscript *R* is the relative space mileage relative to $S_{Ref}$; $S_{i}$ is the location of the guideway inspection equipment at time *i*, $S_{i}^{R}$ is the relative space mileage, $S_{i}^{A}$ is the absolute space mileage; $G_{j}$ and $T_{k}$ are the serial numbers of the track beam and stator tooth in the beam at time *i*. Thus, the absolute position $S_{i}^{A}$ of the guideway inspection equipment at time *i* can be described as:

(1) $$\begin{array}{cc} S_{i}^{A}= & S_{Ref}^{A}+S_{i}^{R}\\ = & S_{Ref}^{A}+S_{G_{i}}^{R}+S_{T_{i}}^{R}+S_{P_{i}}^{R} \end{array}$$

where $S_{G_{i}}^{R}$ is the sum of the length of the completely passed track beams from the reference point $S_{Ref}$, $S_{T_{i}}^{R}$ is the length of the completely passed stator teeth in the current track beam, and $S_{P_{i}}^{R}$ is the mileage passed by the guideway inspection equipment in the current tooth-slot cycle.

Formula (1) is further converted to the tooth-slot cycle $L_{T}$, and the stator-tooth-slot-arrangement-based locating algorithm is obtained, as shown in formula (2):

(2) $$\begin{array}{cc} S_{i}^{A}= & S_{Ref}^{A}+\overset{j-1}{\underset{i=0}{\sum }}T_{G_{i}}\times L_{T}+\overset{k-1}{\underset{i=0}{\sum }}L_{T}+\overset{l}{\underset{i=0}{\sum }}\frac{L_{T}}{R_{k+1}-R_{k}}\\ = & S_{Ref}^{A}+L_{T}\times \left(\overset{j-1}{\underset{i=0}{\sum }}T_{G_{i}}+\overset{k-1}{\underset{i=0}{\sum }}1+\overset{l}{\underset{i=0}{\sum }}\frac{1}{R_{k+1}-R_{k}}\right) \end{array}$$

where subscript *j* is the relative serial number of the track beam; subscript *k* is the tooth number in the current beam; subscript *l* is the sample number in the current tooth-slot cycle; $T_{G_{i}}$ is the number of stator teeth inside the track beam $G_{i}$; $R_{k}$ and $R_{k+1}$ are the sample numbers of the rising edge of the *k*th and (*k* + 1)th tooth-slot cycle in the beam, respectively.

## 4. Processing of the Sensor Signal

**Figure 7:**
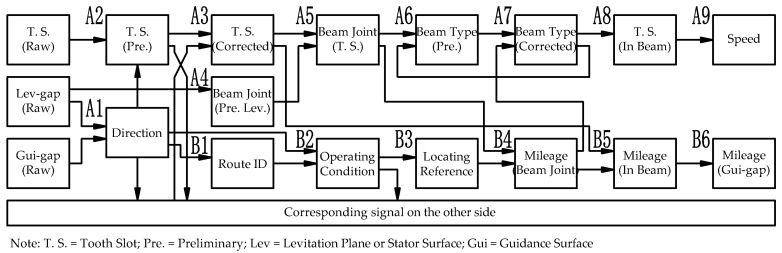
Locating algorithm of guideway inspection data (single side).

According to the characteristics of the sensor signal collected by guideway inspection equipment and the locating principle, the speed measurement and the locating algorithm is designed, as shown in Figure 7:

### 4.1. Obtaining the Speed Information

According to Table 2, the speed information mainly comes from the tooth-slot signal, but the tooth-slot signal contains random interference near the beam joint. Therefore, obtaining an accurate tooth-slot signal and accurate beam joint position are keys to realizing speed measurement and positioning.

**Figure 8:**
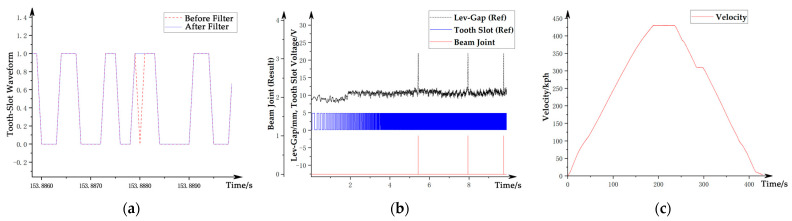
Processing of the velocity signal: (**a**) tooth-slot signal before and after filtering; (**b**) beam joint recognized; (**c**) velocity recognized.

Referring to Figure 7, and taking the left side as an example, the main steps for obtaining the speed information are as follows:

(A1) Identify the running direction based on the phase difference between the preliminary beam joint pulse extracted from the guidance gap signal and the levitation gap signal;
(A2) Obtain the preliminary tooth-slot signal by reshaping the raw tooth-slot data with parameters identified from the running direction;
(A3) Fuse the preliminary tooth-slot signals on both left and right sides and perform anti-interference filtering. The method is to observe the corresponding width and change rate of the tooth surface of the signal on both sides at the same time. Divide the signal quality into different situations as noninterference, single-side interference, and double-side interference. Then, process them separately to obtain the corrected tooth-slot signal without interference, as shown by the blue line in Figure 8a;
(A4) Extract the preliminary beam joint position of the stator surface from the levitation gap signal;
(A5) Fuse the preliminary beam joint position and the corrected tooth-slot signal to obtain the accurate beam joint position synchronized with the tooth-slot phase at the stator surface, which is called the tooth-slot beam joint, as shown by the red line in Figure 8b;
(A6) Locate the accurate beam end position based on the tooth-slot beam joint information on the stator surface, and calculate the type (representing the length) of the track beam by counting the corrected teeth within the track beam;
(A7) Check and correct the beam type by comparing the length with the accurate length obtained from the positioning analysis in step (B4);
(A8) Check and correct the tooth-slot recognition results in the beam range using the accurate beam length on the stator surface;
(A9) Calculate the velocity through accurate tooth-slot identification results; the final vehicle velocity curve obtained is shown in Figure 8c.

### 4.2. Obtaining the Spatial Mileage Information

**Figure 9:**
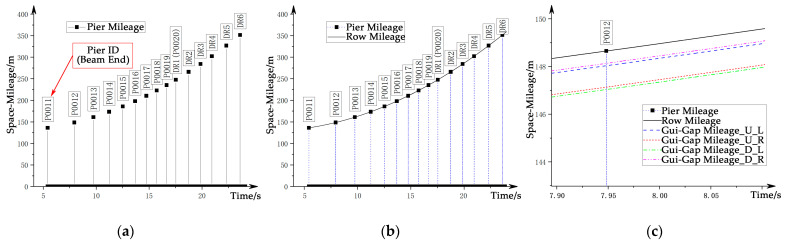
Processing of the locating signal: (**a**) locating result at beam ends; (**b**) locating results inside beams; (**c**) locating results for guidance-gap.

Referring to Figure 7, taking the left side as an example, the main steps for obtaining the spatial mileage information are as follows:

(B1) Identify the characteristic track structures such as turnouts by using the direction information identified in step (A1) and the preliminary beam joint position identified in step (A4) to obtain the train’s route ID;
(B2) Obtain the operating condition information through the combination of the operating track and the operating direction information;
(B3) Obtain the absolute space mileage of the characteristic reference point ($S_{Ref}^{A}$) of the corresponding route ID from the route database;
(B4) Obtain the absolute space mileage at the beam end position, the beam type, and the pier ID by combining the relative space mileage ($S_{G_{i}}^{R}$) of the tooth-slot beam joint with the position of the reference point. The information is calibrated with the design value, and the result is shown in Figure 9a;
(B5) Calculate and calibrate the absolute space mileage ($S_{i}^{A}$) within each beam using information such as the corrected tooth-slot signal, the absolute position of the beam end, and the running direction information; the result is shown in Figure 9b;
(B6) Calculate the corresponding absolute space mileage for each guidance gap channel by the obtained space mileage on stator surface, as shown in Figure 9c.

### 4.3. Domain Conversion of the Data

**Figure 10:**
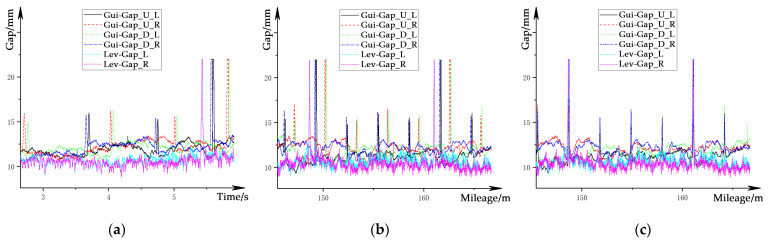
Processing of the gap signals: (**a**) gap signals in time domain; (**b**) gap signals in spatial domain; (**c**) synchronized gap signals in spatial domain.

The conversion converts the speed-sensitive data sampled in the time domain, as shown in Figure 10a, into the spatial domain and synchronizes the data from sensors installed in different longitudinal measurement sections based on the space mileage, to obtain the speed-independent signal based on a unified longitudinal measurement section. The main steps are as follows:

(1) Calculate the absolute space mileage for each piece of tooth-slot data in the stator surface as a reference based on the calibrated tooth-slot beam joint data;
(2) Based on the reference of the tooth-slot data, the space mileage of the levitation gap sensor and the guidance gap sensors at each sample are respectively calculated, and the speed-independent gap signals in the spatial domain are obtained, as shown in Figure 10b;
(3) Calibrate the signal from each gap sensor separately with the absolute space mileage as a unified scale to realize the signal synchronization for all channels. The synchronized gap signals are shown in Figure 10c.

## 5. Results

**Figure 11:**
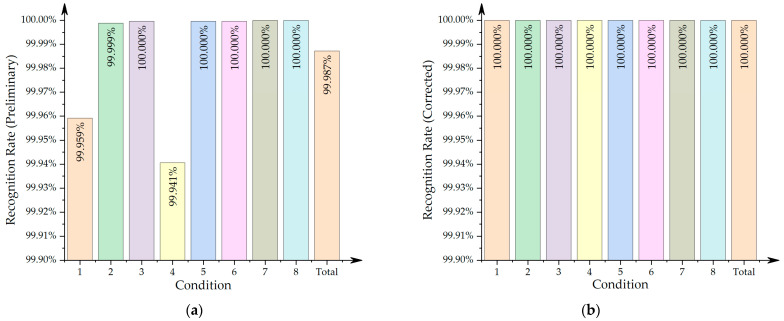
Recognition rate of the teeth slot: (**a**) preliminary result; (**b**) final result (corrected).

In order to verify the correctness of the locating results, a total of four sets of whole-process test data with different running directions and different running tracks were selected for analysis. In each set, the left and right sides were calculated separately. Thus, there were a total of eight sets of data with different working conditions. The beam joint recognition result takes the position deviation within one tooth-slot cycle as the recognition success. The tooth-slot recognition error is the difference between the recognized quantity and the theoretical quantity. The denominator in each percentage calculation is the theoretical value. The summarized identification results of beam joints and teeth are shown in Table 3 and Figure 11:

As shown in Table 3, the recognition rate of the track beam joint under different working conditions was 100%. The recognition rate of the tooth-slot signal on the stator surface before fusion varied according to the operating conditions, with an average recognition rate of about 99.987%, as shown in Figure 11. Among them, under the most unfavorable conditions (condition 4), there were 201 error teeth on the right side, which accounted for about 0.06% of the theoretical teeth (338,480 in total), that is, the lowest recognition accuracy rate was about 99.94%. After the fusion process, all abnormal teeth were removed, achieving a 100% tooth-slot recognition rate. All the guideway inspection data achieved a locating accuracy of stator tooth-slots.

## 6. Discussion

The results from real vehicle data show that even under the unfavorable working conditions where the tooth-slot signal is interfered, the final recognition rate corrected by the algorithm still reached 100%. It fully illustrates the effectiveness, accuracy, and stability of this algorithm under the high-speed operation of maglev trains.

During the analysis, the author found that the recognition rate of the preliminary tooth-slot signal obtained in step A2 showed the following characteristics. Although this phenomenon did not affect the final positioning result, it reflects that the quality of the preliminary tooth-slot signal was different under different operating conditions:

(1) The recognition rate is related to the running track of the train. The tooth-slot recognition rate of track B is significantly higher than that of track A. This may be due to the arrangement of the stator pack and the mileage of the route being based on track B in the design;
(2) The recognition rate of track A is related to the longitudinal direction and the lateral direction at the same time. Although the definitions of the left and right sides of the equipment and track are not changed under each working condition, the results show that the data quality on the right side is significantly better than that on the left side when running in the forward direction, while the opposite is true when running in the reverse direction. It is still unable to explain the reasons; thus, it needs further attention in future research.

## 7. Conclusions

This research presents a locating method for maglev guideway inspection equipment based on inspection data. This method extracts the characteristic information on the track structure from the levitation and guidance gap sensor signals that are perpendicular to the train running direction to obtain the relative and absolute positions of the train. For the first time, this method realizes the function independently and accurately obtains the position without using external hardware corrections such as OCS locating sensors, LRF information, onboard vehicle network data, and GPS. The locating accuracy is better than the one tooth-slot cycle (86 mm) in the whole process. Compared with traditional locating methods for track inspection equipment, the advantages of this method are:

(1) Higher absolute locating accuracy. Compared with the traditional method of accumulating the relative position sensor signal and correcting with the LRF signal, which usually takes about 15 beams (approximately 370 m, depending on the arrangement of the LRF) to clear the accumulated error of the beam end (there may be a small amount of accumulated error of mileage between two LRFs), this method automatically corrects the absolute position of the beam end for each beam, so there is no cumulative error in the whole journey;
(2) Higher relative locating accuracy. Compared with the traditional method with the GPS receiver and other third-party hardware that can only locate the track beam roughly, this method can obtain a locating accuracy better than that of the long stator tooth-slot period (86 mm), thereby enabling a better pertinence of track inspection;
(3) Higher train speed compatibility. As the locating accuracy is not affected by the speed of the maglev train, not only can this method be used in the current 430 km/h maglev track inspection system, but it can also be applied to the next generation 600 km/h or higher maglev track inspection fields with the same accuracy;
(4) With operating condition automatic recognition. Compared with the traditional method that requires additional hardware to obtain operating condition information such as the direction, track ID, and maximum speed from the on-board CAN bus, this method can automatically extract the aforementioned information directly from the data of the track inspection sensor, reducing the hardware requirements for track inspection equipment;
(5) High independence of track inspection equipment. Compared with the traditional method, which needs special hardware channels for vehicle network and OCS locating sensors, this method directly analyzes the track inspection data to obtain the locating information, which effectively improves the independence of the track inspection equipment.

As maglev transportation technology improves by leaps and bounds, the new generation of 500–600 km/h high-speed maglev approaches commercial use, and tube maglev of over 1000 km/h is being extensively studied worldwide, maglev track inspection equipment will play an increasingly important role. This method has been applied in the data processing of portable high-speed maglev track inspection equipment. The next stage of research will further explore the efficiency optimization of the algorithm, in order to provide more efficient and accurate locating information for independent and miniaturized guideway inspection equipment for high-speed maglev in future.

## Figures and Tables

**Table 1:** Summary of sensor signals.

Serial Number	Signal Name	Signal Type	Source Sensor	Sensor Position	Corresponding Track Surface
1	Levitation Gap Left	Linear Displacement	Levitation Gap Sensor	Levitation Electromagnet	Left Stator Surface
2	Levitation Gap Right	Linear Displacement	Levitation Gap Sensor	Levitation Electromagnet	Right Stator Surface
3	Guidance Gap Left Upper	Linear Displacement	Guidance Gap Sensor	Guidance Electromagnet	Upper Left Guidance Surface
4	Guidance Gap Right Upper	Linear Displacement	Guidance Gap Sensor	Guidance Electromagnet	Upper Right Guidance Surface
5	Guidance Gap Left Lower	Linear Displacement	Guidance Gap Sensor	Guidance Electromagnet	Lower Left Guidance Surface
6	Guidance Gap Right Lower	Linear Displacement	Guidance Gap Sensor	Guidance Electromagnet	Lower Right Guidance Surface
7	Tooth-slot signal Left	Square Wave Pulse	Levitation Gap Sensor	Levitation Electromagnet	Left Stator Surface
8	Tooth-slot signal Right	Square Wave Pulse	Levitation Gap Sensor	Levitation Electromagnet	Right Stator Surface

**Table 2:** Summary table of speed and positioning information contained in signals.

No.	Items	Lev-Gap Signal	Gui-Gap Signal	Tooth-Slot Signal	Remarks
1	Speed info.	Include indirectly	Include indirectly	Include but interfered	
2	Relative locating info.	Include but interfered	Include but interfered	Include but interfered	
3	Locating resolution	Track beam	Functional component	Stator tooth	Ideal signal
4	Position phase difference	No	Different for each channel	No	
5	Absolute locating info.	Include indirectly	Contain indirectly	No	
6	Beam joint info.	Contain but interfered	More interfered	No	

**Table 3:** Summary of locating calculation results.

SN	Dir.	Trk.	Side	Samples /Row	Beam Joints Ident.			Teeth Ident. (Preliminary)				Teeth Ident. (Corrected)			
					Theo. Qty. /pcs	Ident. Qty. /pcs	Recon. Rate /%	Theo. Qty. /T	Ident. Qty. /T	Error Qty. /T	Recon. Rate /%	Result. Qty. /T	Corr. Qty. /T	Error Qty. /T	Recon. Rate /%
1	+	A	L	4,420,000	1184	1184	100	338,482	338,620	138	99.959	338,482	138	0	100
2	+	A	R	4,420,000	1184	1184	100	338,482	338,486	4	99.999	338,482	4	0	100
3	-	A	L	4,450,000	1184	1184	100	338,480	338,481	1	100.000	338,480	1	0	100
4	-	A	R	4,450,000	1184	1184	100	338,480	338,681	201	99.941	338,480	201	0	100
5	+	B	L	4,900,000	1181	1181	100	338,607	338,608	1	100.000	338,607	1	0	100
6	+	B	R	4,900,000	1181	1181	100	338,607	338,608	1	100.000	338,607	1	0	100
7	-	B	L	4,920,000	1181	1181	100	338,607	338,607	0	100	338,607	0	0	100
8	-	B	R	4,920,000	1181	1181	100	338,607	338,607	0	100	338,607	0	0	100
	Total			37,380,000	9460	9460	100	2,708,352	2,708,698	346	99.987	2,708,352	346	0	100

Note: Theo. = theoretical; Ident. = identified; Recon. = recognition; T = tooth; Corr. = corrected.

## Data Availability

The data presented in this study are available on request from the corresponding author with permission. The data are not publicly available, due to participant confidentiality.
